# Monte Carlo Simulation of Clothed Skin Exposure to Electromagnetic Field With Oblique Incidence Angles at 60 GHz

**DOI:** 10.3389/fpubh.2022.795414

**Published:** 2022-02-14

**Authors:** Kun Li, Kensuke Sasaki

**Affiliations:** ^1^Faculty of Engineering and Design, Kagawa University, Takamatsu, Japan; ^2^Radio Research Institute, National Institute of Information and Communications Technology, Koganei, Japan

**Keywords:** electromagnetic fields, millimeter wave, human skin, cloth effect, absorbed power density, exposure guideline

## Abstract

This study presents an investigation of clothed human skin exposure to obliquely incident electromagnetic waves at 60 GHz. We clarified the combined impacts of the cloth material, incidence angle, and polarization on the assessment of transmittance and absorbed power density (*APD*) at the skin surface. A Monte Carlo simulation was conducted considering the thickness variation of the cloth material and skin tissue. For the case of transverse magnetic™ wave exposure, the transmittance increases with increasing incident angle up to the maximum transmittance angle in the range from 60 to 80°, which is known as the Brewster effects, regardless of textile materials and air gap between cloth and skin. The air gap results in a periodic fluctuation of the *APD*, where the variation is almost within 1 dB when the incident power density is constant and the incident angle is smaller than 40°. Our results also show that as the air gap increases to 2.5 mm, i.e., half-wavelength at 60 GHz in the air, the *APD* within the skin surface covered by typical cloth materials increases up to 40% compared with that of bare skin. Although the use of several cloth materials may increase the transmittance and *APD* in oblique incidence scenarios, all the results of the *APD* do not exceed the basic restriction for local exposure, demonstrating that the current guidelines for human exposure to electromagnetic fields are appropriate for preventing the excessive exposure at 60 GHz considering the impacts of oblique incidence angles and cloth materials.

## Introduction

In the upcoming beyond 5G/6G wireless communication system, millimeter-wave (MMW) devices have received considerable attention owing to the potentially high data rate transmission and a large amount of available bandwidth (1, 2). The increasing demand for MMW radio frequency (RF) transmitters operated in the human vicinity, such as mobile phones, tablet terminals, and Wi-Fi systems, has raised public concerns about human exposure to electromagnetic fields (EMFs) (3–7). The International Commission on Non-Ionizing Radiation Protection (ICNIRP) guideline (8) and the Institute of Electrical and Electronics Engineers (IEEE) International Commission on Electromagnetic Safety (ICES) (IEEE Standard C95.1) (9) have recommended the absorbed power density (*APD*) or epithelial power density, respectively, as a new metric for the basic restriction (BR) or dosimetric reference limit (DRL) to protect against the adverse health effects associated with superficial heating for local exposures at frequencies from 6 to 300 GHz. The absorbed or epithelial power densities crossing a unit area in the direction normal to the body interface represents the total power deposited in the biological tissues, which was derived from an operational health effect threshold in terms of the temperature rise divided with the reduction factors employed in the RF exposure guidelines and standards (8, 9). According to the BR/DRL, exposure limits of incident power density (*IPD*) used as reference level (RL) (8) or exposure reference levels (ERLs) (9) were derived. For local exposures at a frequency from 6 to 300 GHz, the *IPD* should not exceed 275$f_{G}^{-0.177}$ and 55$f_{G}^{-0.177}$ (W/m^2^) (*f*_*G*_: frequency in GHz) for occupational exposure/restricted environments and general public exposure/unrestricted environments, respectively (8, 9).

Recent dosimetric studies to electromagnetic field exposure at MMW bands mainly aim to clarify the relationship between different definitions of power densities and temperature rise at skin surface including both plane wave incidence and practical RF sources (10–27). The first concern in investigating these subjects is the consideration of oblique radio wave incidence in general human exposure scenarios, especially referred to as beam steering technology, employed in 5G wireless systems. The incident angle dependence of transmittance, *APD*, and skin temperature elevation has been studied for plane-wave exposures (12–14) where the difference for transverse electric (TE) and transverse magnetic (TM) plane waves injection such as Brewster' angle effect was clarified. He et al. (24) and Nakae et al. (25) reported the research results of exposures to oblique incidence electromagnetic fields from phased array antennas at 28 GHz, i.e., the FR2 (MMW) frequency band assigned in Japan and some other countries. Considering the extension to other MMW bands, a new working group 5 under Subcommittee 6 of IEEE ICES TC95 was established to clarify these aspects, where the effect of the incidence angle on the spatial-average power densities and resultant temperature elevation using both computational and thermographic measurement approaches were reported (26, 27). It was found that the normal incidence scenario is generally the worst-case for surface temperature rise when bare skin is directly illuminated by an electromagnetic field.

Another important topic is the consideration of cloth effects for more realistic human exposure conditions. During the use of MMW wireless device approaching a human body wearing clothes made of different textile materials, the difference compared with bare skin should be clarified for accurate dosimetry. Several studies have investigated the cloth impacts on the variation of electromagnetic power deposition at MMW (3, 28–32). Through the analysis of the power penetration, it is well-known that the cloth material acts as an impedance transformer and may increase the power absorption in the clothed skin. In addition, the impact of a textile layer in the contact or proximity of skin on the power transmission coefficient, *APD* and temperature rise at 26 and 60 GHz has been reported in the literature (31). The literature (31) firstly gives a detailed evaluation of power absorption and thermal dosimetry under plane wave normal incidence conditions considering the influence of the textile material and air gap spacing. They clarified that the presence of an air gap between the cloth and the skin modifies the electromagnetic power deposition, which may result in a temperature rise variation from −11.1 to 20.9% compared to the bare skin at 60 GHz. However, the evaluation of power deposition at oblique incidence has not been investigated sufficiently. Considering a general exposure environment, it is essential to assess the clothed skin dosimetry for cases of not only normal incidence but also oblique incidence. The transmittance and *APD* may be significantly different from those of bare skin, which will be determined as the combined outcome of cloth materials, incident angles, polarization components, and air gap spacing.

In this study, we aim to analyze the electromagnetic field exposure of a clothed human skin tissue with an obliquely incident plane wave at 60 GHz. Following previous work, dosimetric studies were conducted by a theoretical analysis using the biological-tissue parameters employed by (15, 33). Variations of the transmittance and the *APD* were evaluated considering the dispersion of cloth and skin tissue thickness by Monte Carlo simulation. The effects of the air gap between the cloth and the skin were examined considering various textile materials, incidence angles, and polarization.

## Method and Model

**Figure 1:**
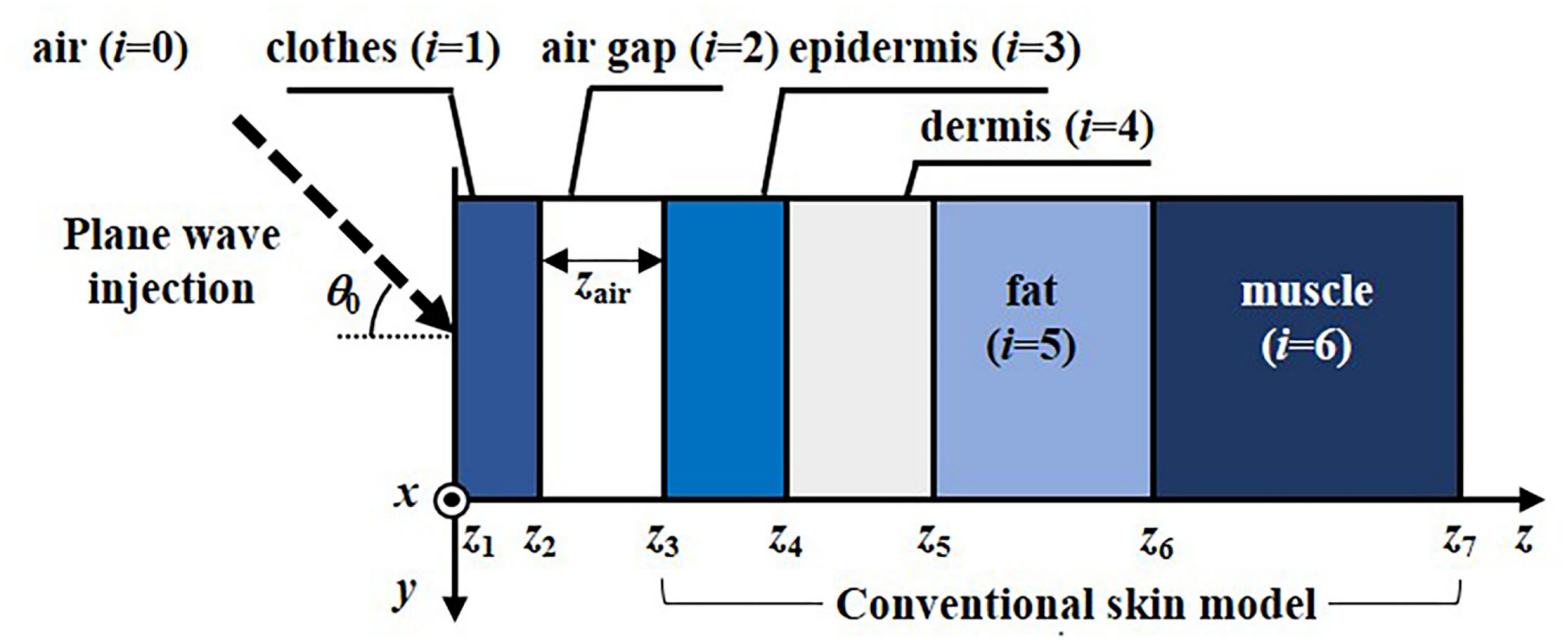
Two-dimensional clothed skin model composed of various cloth material (*i* = 1), air gap layer (*i* = 2), epidermis (*i* = 3), dermis (*i* = 4), fat (*i* = 5), and muscle (*i* = 6) exposed to an obliquely incident plane wave.

Figure 1 illustrates a two-dimensional analytical model composed of cloth material, air gap space, and a conventional multi-layer human skin tissue. The skin model consists of the epidermis, dermis, subcutaneous fat, and muscle used to represent the skin tissue in the abdomen (15). Six textile materials used for typical cloth manufacturing were employed, which are cotton (M1), wool (M2), linen (M3), leatherette (M4), polyester fiber (M5), and latex mattress (M6), respectively. An oblique incident plane wave injected from the air to the surface of the cloth material layer with an angle of θ_0_ is assumed. Two polarization components of the incident waves are considered individually, defined as TE and TM waves, whose electric-field vectors are perpendicular and parallel to the incident plane (*yz*-plane), respectively. The incident power density of 26.6 W/m^2^, i.e., the reference level for local exposure of the general public at 60 GHz, as indicated in ICNIRP 2020 (8) and IEEE C95.1 2019 (9). The incident power density is defined at the plane whose normal is parallel to the wave-number vector. The *APD* crossing a unit area at *z* = *z*_3_ in the air gap to skin boundary in the direction normal to the interface (14, 17, 18), is given by,


(1) $$\begin{array}{r} APD\;=\;{\frac{1}{2}R\left\{\left(E\left(z\right)\times H^{*}\left(z\right)\right)•n\right\}|}_{z=z_{3}} \end{array}$$

where ***E*** and H^*^ denote the electric field phasor and the complex conjugate of the magnetic field phasor, respectively. Table 1 listed the parameters of cloth materials and skin tissues in the Monte Carlo simulation. For consistency with the results obtained by (14), the dielectric properties of multi-layer skin tissue reported by (15, 33) were used. The mean values and standard deviations of various tissue thicknesses of the abdomen [see Table 2 in (15)] were used, which was measured by (34, 35) using a sufficient number of samples considering the individual differences in gender and age. In addition, we employed the relative complex permittivities and thicknesses of six different types of cloth materials based on the published data in (31, 36). The Monte Carlo simulation was conducted based on the statistical data on cloth material and skin tissue thickness using normally distributed random numbers generated by Matlab R2021a (14). The number of iterations was set to 10^4^ for each oblique incidence angle and air gap spacing.

**Table 1:** Dielectric constants and thickness of cloth materials and skin tissues (mean ± standard deviation) used in Monte Carlo simulation.

**Model**	**Layer**	**Thickness (mm)**	**Relative complex permittivity**
		**(mean ±std)**	** $\left(\varepsilon_{r}=\varepsilon_{r}{}^{\prime }-j\varepsilon_{r}{{}^{\prime }}^{\prime }\right)$ **
Cloth	Cotton (M1)	0.2 ± 0.02	2.0 −*j*0.04
	Wool (M2)	2.0 ± 0.02	1.22 −*j*0.036
	Linen (M3)	0.98 ± 0.1	1.25 −*j*0.102
	Leatherette (M4)	0.91 ± 0.007	2.16 −*j*0.021
	Polyester Fiber (M5)	0.79 ± 0.194	1.22 −*j*0.003
	Latex Mattress (M6)	11.34 ± 0.241	1.11 −*j*0.009
Skin	Epidermis	0.0794 ± 0.0339	8.5 −*j*9.9
	Dermis	1.25 ± 0.26	10.4 −*j*11.9
	Fat	14.3 ± 7.5	5.7 −*j*4.7
	Muscle	14.4 ± 3.5	10.7 −*j*13.9

## Results

### Transmittance and Absorbed Power Density at Skin Surface

**Figure 2:**
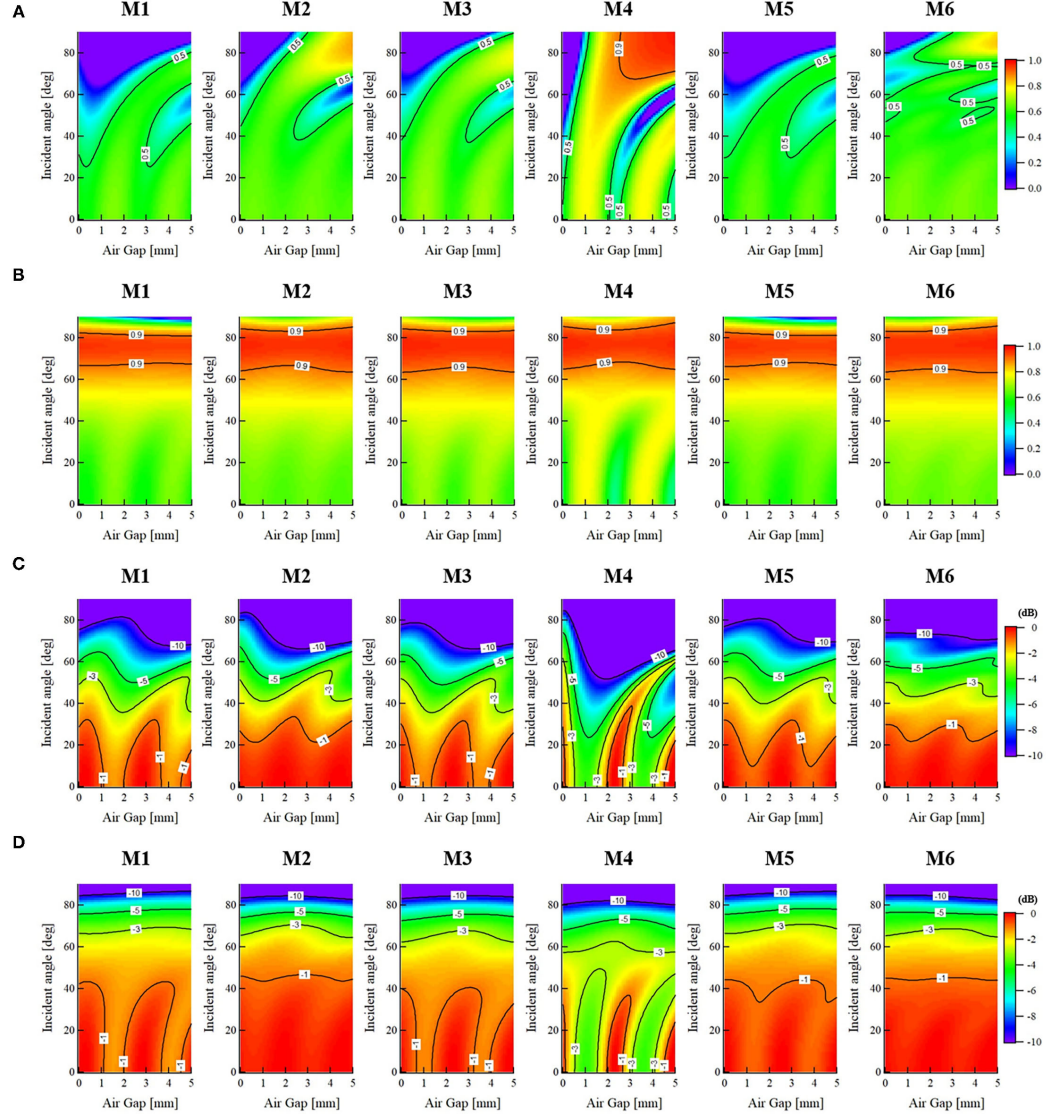
Mean values of transmittance and absorbed power density at skin surface as functions of oblique incidence angles θ_0_ and air gap spacing *z*_air_ when the skin model is covered by various types of cloth materials at 60 GHz, **(A)** Transmittance (TE wave), **(B)** Transmittance (TM wave), **(C)** *APD* (TE wave), and **(D)** *APD* (TM wave).

Figures 2A,B show the transmittance as functions of incident wave angle (θ_0_) and air gap spacing (*z*_air_) when the skin is covered by cloth materials. Figure 2A shows the results for TE waves, whereas Figure 2B shows those for the TM waves. The mean value of transmittance at the skin surface is defined as,


(2) $$T\;=\;1-{\left|\gamma_{2}\left(\theta_{2}\right)\right|}^{2}$$

where γ_2_ denotes the reflection coefficient from the skin surface to the air gap. θ_2_ indicates the incident wave angle from the air gap layer to the skin surface, which equals θ_0_ in the air on the basis of Snell's law.

In Figure 2A, the transmittance of the TE waves in each cloth material decrease with increasing incident wave angle. For the incident angle θ_0_ smaller than 40°, most of the mean transmittance are shown below 0.5. When the cloth material of M4 is used, a relatively obvious fluctuation with the variation of air gap spacing is observed in this range of incident angles. Moreover, when the air gap is larger than 2.5 mm and the incident angle θ_0_ is higher than 70°, a significant increase of transmittance over 0.9 is observed. This is different from the general characteristics of the TE waves incidence on bare skin. The reason may be attributed to the function of the impedance transformer of the cloth material (M4) affected by its dielectric property and thickness, which may result in a complicated phase reversal phenomenon with the increase in the air gap spacing.

On the other hand, the TM waves show increased transmittance with increasing θ_0_ up to the maximum transmittance angle, which is known as the Brewster effect. In Figure 2B, for each cloth material, the maximum transmittance angle for the TM waves varies from about 60 to 80° regardless of the variation of air gap spacing. This fact corresponds well with that of bare skin at 60 GHz (14), indicating that the cloth material and air gap do not significantly affect the transmittance at the skin surface when the clothed skin is exposed to TM waves injection. In addition, similar to the behavior of TE waves, the dynamic variation using the cloth material of M4 at small incidence angles is relatively larger compared with other textile materials.

Figures 2C,D indicate the *APD* normalized to the maximum value as functions of incident wave angle (θ_0_) and air gap spacing (*z*_air_) considering the cloth effects. The contour lines in the figures show the difference from the maximum mean value of the *APD* at the skin surface. Figure 2C shows the results for TE waves, whereas Figure 2D shows those for TM waves. The mean value of *APD* within the skin surface is obtained by **Equation 1**.

In Figure 2C, the *APD* of the TE waves in each cloth material decrease with increasing incident wave angle. For the incident angle θ_0_ smaller than 40°, an oscillatory behavior as the increase of air gap spacing is observed. Especially when the cloth material of M4 is used, a significant fluctuation with a reduced period is shown compared with other cloth materials. This indicates that the variation of an air gap between the cloth and skin can decrease or increase the electromagnetic field power deposition in the skin tissues, as reported in (31). However, the variation normalized to the maximum mean value of *APD* is within −1 dB when θ_0_ <40°. With increasing the incident wave angle, the dynamic range from a peak to the valley is reduced, where the degradation of *APD* is almost below −5 and −10 dB, respectively, when θ_0_ is <60 and 80°. Among all the textile materials, the relative standard deviation of the *APD* is within 6.9, 9.7, and 26.5% when the oblique incidence angle is smaller than 40, 60, and 80°, respectively.

For the TM waves, as shown in Figure 2D, the *APD* for all the cases of cloth materials also decreases with increasing incident wave angle, indicating that the normal incidence is also the worst-case exposure condition even for a clothed skin when an air gap spacing is determined. For the incident angle θ_0_ <40°, an oscillatory behavior as the increase of air gap can be observed only in the cloth materials of M1, M3, and M4. For those using M2, M5, and M6, the dynamic behavior is relatively small. Similar to the TE waves, a severe fluctuation with a reduced period is shown when the cloth material of M4 is used compared with other cloth materials. The variation normalized to the maximum mean value of *APD* is also within −1 dB when θ_0_ <40°. With the increase of θ_0_, both the entire level and the fluctuation of the *APD* obviously reduce. Particularly, for TM wave incidence at θ_0_ > 60°, the *APD* within the skin surface shows an almost flat profile with the increase of air gap, indicating that the increase of oblique incidence angle will reduce the variation of the *APD* due to the air gap effects. Moreover, the relative standard deviation of the *APD* is within 5.9, 5.1, and 7.6% when the incidence angle is smaller than 40, 60, and 80°, respectively. Thus, it was found that the contribution of the variation in cloth and skin thickness to the *APD* is not significant.

### Comparison of Transmittance and Absorbed Power Density With Bare Skin

**Figure 3:**
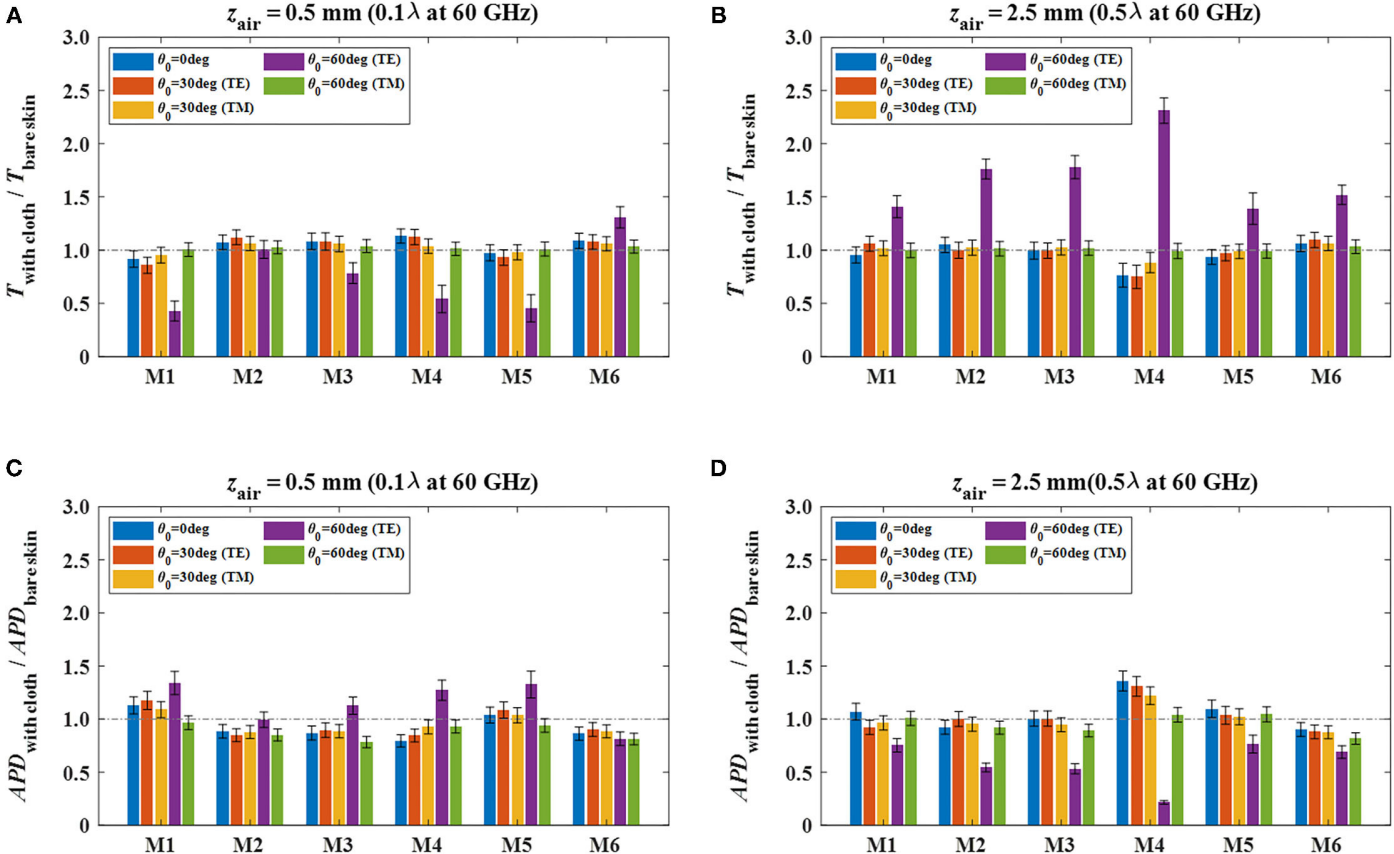
Ratios of transmittance and absorbed power density at skin surface with various cloth materials to those of bare skin at different air gap spacing, **(A)** *T*_with cloth_/*T*_bare skin_ (*z*_air_ = 0.5 mm), **(B)** *T*_with cloth_/*T*_bare skin_ (*z*_air_ = 2.5 mm), **(C)** *APD*_with cloth_/*APD*_bare skin_ (*z*_air_ = 0.5 mm), and **(D)** *APD*_with cloth_/*APD*_bare skin_ (*z*_air_ = 2.5 mm).

Figure 3 shows the comparison results of transmittance and *APD* for the cases between the clothed and bare skin. Figures 3A,B indicate the ratio of mean transmittance with cloth (*T*_with cloth_) to that of bare skin without cloth (*T*_bare skin_) when the air gap spacing (*z*_air_) is set to 0.5 and 2.5 mm, respectively. Figures 3C,D indicate the ratio of mean *APD* with cloth (*APD*_with cloth_) to that of bare skin (*APD*_bare skin_) when the *z*_air_ is set to 0.5 and 2.5 mm, respectively. The error bars denote the standard deviations of the *APD* by the Monte Carlo simulation, which also implies the variation of *APD* due to the change of the thickness of cloth and skin tissue.

In Figure 3A, when *z*_air_ = 0.5 mm, which equals 0.1λ at 60 GHz (λ: free space wavelength), the results of *T*_with cloth_ and *T*_bare skin_ are almost comparable with each other. The maximum ratio of *T*_with cloth_ to *T*_bare skin_ of 1.3 occurs when the clothed skin with textile material of M6 is exposed by a TE wave with an incidence angle of 60°. For cases of other materials, the corresponding ratio, i.e., TE wave injection at θ_0_ = 60°, reduces to <1.0. When *z*_air_ increases to 2.5 mm, which equals 0.5λ at 60 GHz, all the results do not exceed 1.0 obviously except for the cases of TE wave injection at θ_0_ = 60°, as shown in Figure 3B. In that case, the maximum ratio of *T*_with cloth_ to *T*_bare skin_ exceeds 2.3 when the cloth material of M4 is used. In addition, the relative standard deviations for cases of cloth materials are below 28.2 and 14.7%, respectively, when *z*_air_ is 0.1 and 0.5 mm.

In Figure 3C, when *z*_air_ = 0.5 mm, most of the results of the *APD*_with cloth_ show equivalent levels or below those of the *APD*_bare skin_. Different from the relationship of transmittance shown in Figure 3A, the maximum ratio of *APD*_with cloth_ to *APD*_bare skin_ of 1.3 occurs when the clothed skin with textile materials of M1 and M5 is exposed by TE waves when θ_0_ = 60°. In contrast, when *z*_air_ increases to 2.5 mm, all the ratios of *APD*_with cloth_ to *APD*_bare skin_ significantly degrades to <0.8 when TE waves incident at θ_0_ = 60°. Instead of this, the maximum ratio of 1.4 occurs at normal incidence when M4 material is used. In addition to the above, other results show that there is no significant difference of the *APD* between the clothed and bare skin when exposed to oblique incidence plane waves at 60 GHz. Moreover, the relative standard deviations for all the cases of cloth materials are within 9.5 and 10.9%, respectively, when *z*_air_ is 0.1 and 0.5λ at 60 GHz, indicating that the effects caused by the variation of cloth thickness to the *APD* is marginal.

## Discussion and Conclusion

This study briefly investigated the EMF exposures of a clothed skin model with an obliquely incident plane wave at 60 GHz. As an extension work of the previous study (14), this work mainly aimed to clarify the combined impacts of the cloth material, incidence angle, and polarization on the assessment transmittance as well as *APD* specified as the BR in ICNIRP guideline for human exposure to EMF. Our results show that for most cloth materials, the variations of oblique incident angle and air gap between cloth and skin may result in a significant fluctuation but a relatively small level of transmittance of the TE wave except for using the cloth material of leatherette (M4). For TM wave incidence, the maximum transmittance increases with increasing incident angle up to 0.9 in the range from 60 to 80°, which is known as the Brewster effect. Although periodic changes are observed at lower incident angles, it can be considered that the transmittance of the TM wave will not be obviously affected by the cloth material and the air gap.

On the other hand, with the increase of air gap spacing between cloth and skin surface, an oscillatory behavior with a peak to valley variation of the *APD* for both the TE and TM waves were observed. This indicates that the existence of an air gap between the cloth and skin can increase or decrease the *APD* at the skin surface (31), where the dielectric properties and thickness of cloth materials under various incident angles and polarizations will affect the role of the impedance transformer. However, the variation normalized to the maximum mean value of *APD* is only within −1 dB when θ_0_ <40°. On the basis of the reduction factor of 2 (about 3 dB) used for deriving the BR from the operational health effect threshold in local exposure above 6 GHz in the ICNIRP guidelines (8), an absolute difference <1 dB is sufficiently small considering the uncertainty of the evaluation. With increasing the incident angle of θ_0_, the entire level and the fluctuation of the *APD* degrade obviously for both the TE and TM waves. The fact indicates that the normal incidence is the worst-case exposure condition for a clothed skin once the air gap between the cloth and skin is determined. In comparison to the bare skin, the use of various cloth materials under various oblique incidence exposure conditions generally increases the *APD* at the skin surface up to about 40%. In addition, the relative combined standard deviations for all the cases of cloth materials are within 9.5 and 10.9%, respectively, when *z*_air_ is 0.1 and 0.5λ at 60 GHz, indicating that the contribution to the *APD* caused by the cloth thickness variation is not significant. Furthermore, all the results of the *APD* do not exceed the BR for local exposure of 20 W/m^2^ when the *IPD* is set to 26.6 W/m^2^, i.e., the reference level for local exposure of the general public at 60 GHz, demonstrating that under the considered conditions, the current guidelines are appropriate for preventing the excessive exposure at millimeter-wave bands including the combined effects of cloth materials, air gap, oblique incidence angle, and polarization. The findings of this study are useful for understanding the appropriate use of BR in practical MMW exposure scenarios.

## Data Availability Statement

The original contributions presented in the study are included in the article/supplementary material, further inquiries can be directed to the corresponding author/s.

## Author Contributions

KL designed and performed all the simulations, analyzed the data, and wrote the paper. KS provided original data of skin model and dielectric properties and reviewed the manuscript. All authors have read and approved the manuscript.

## Funding

This work was partly supported by the Ministry of Internal Affairs and Communications of Japan Grant Number JPMI10001.

## Conflict of Interest

The authors declare that the research was conducted in the absence of any commercial or financial relationships that could be construed as a potential conflict ofinterest.

## Publisher's Note

All claims expressed in this article are solely those of the authors and do not necessarily represent those of their affiliated organizations, or those of the publisher, the editors and the reviewers. Any product that may be evaluated in this article, or claim that may be made by its manufacturer, is not guaranteed or endorsed by the publisher.
